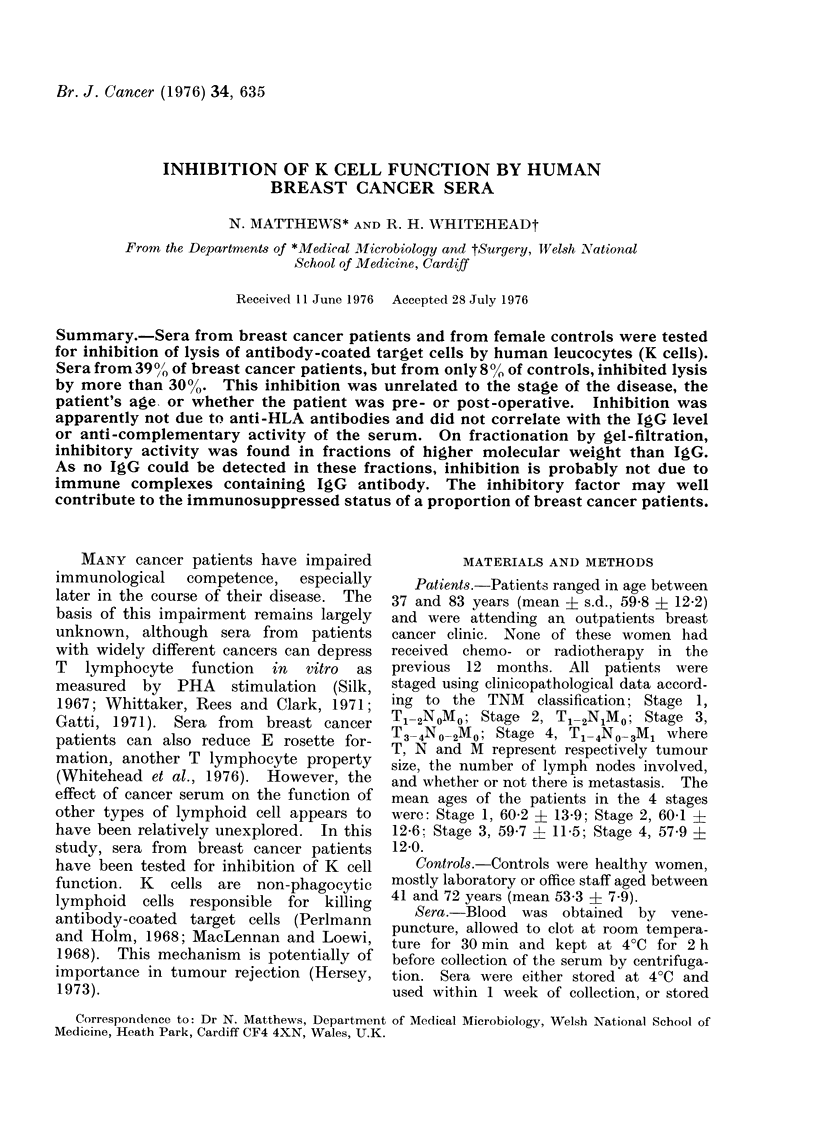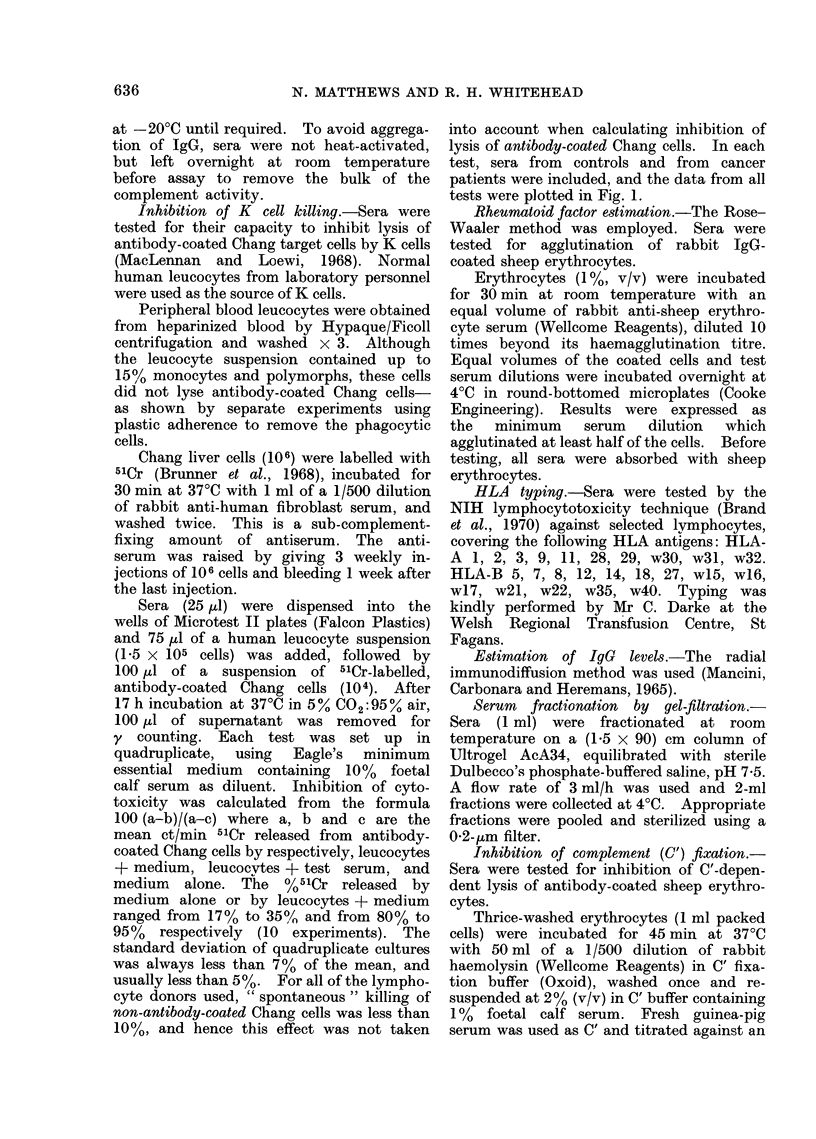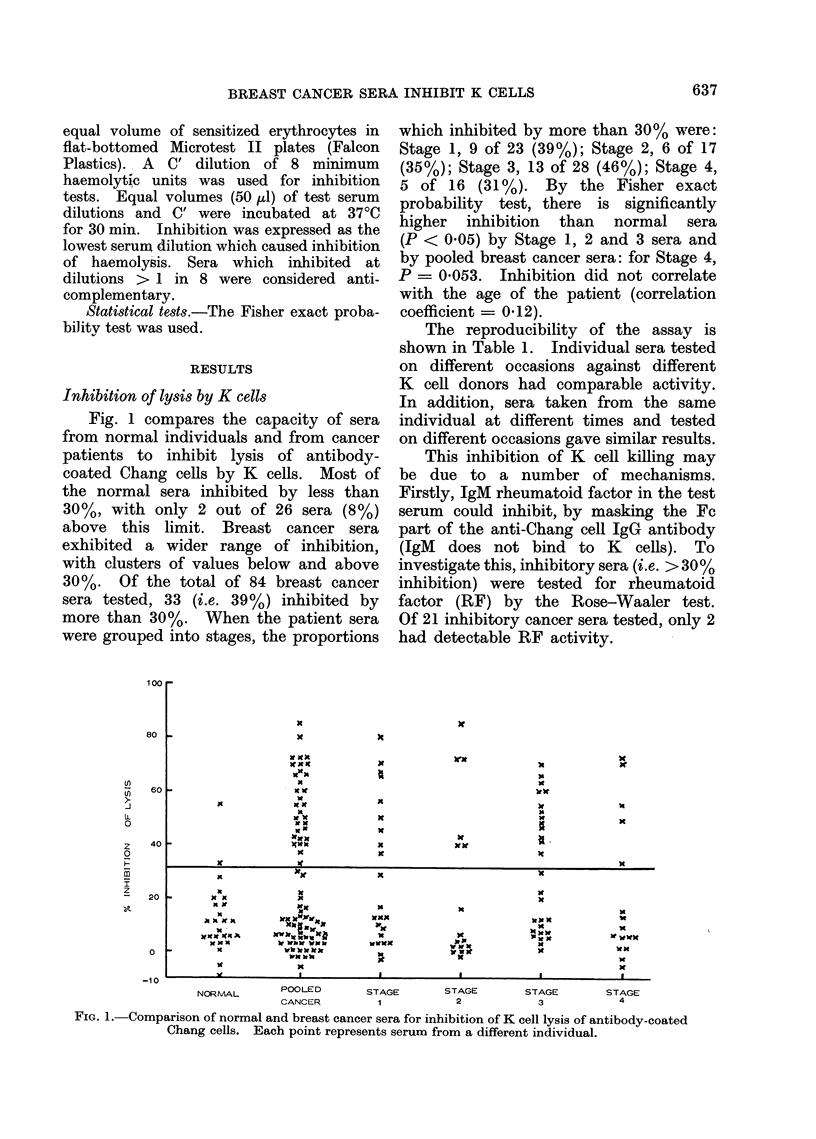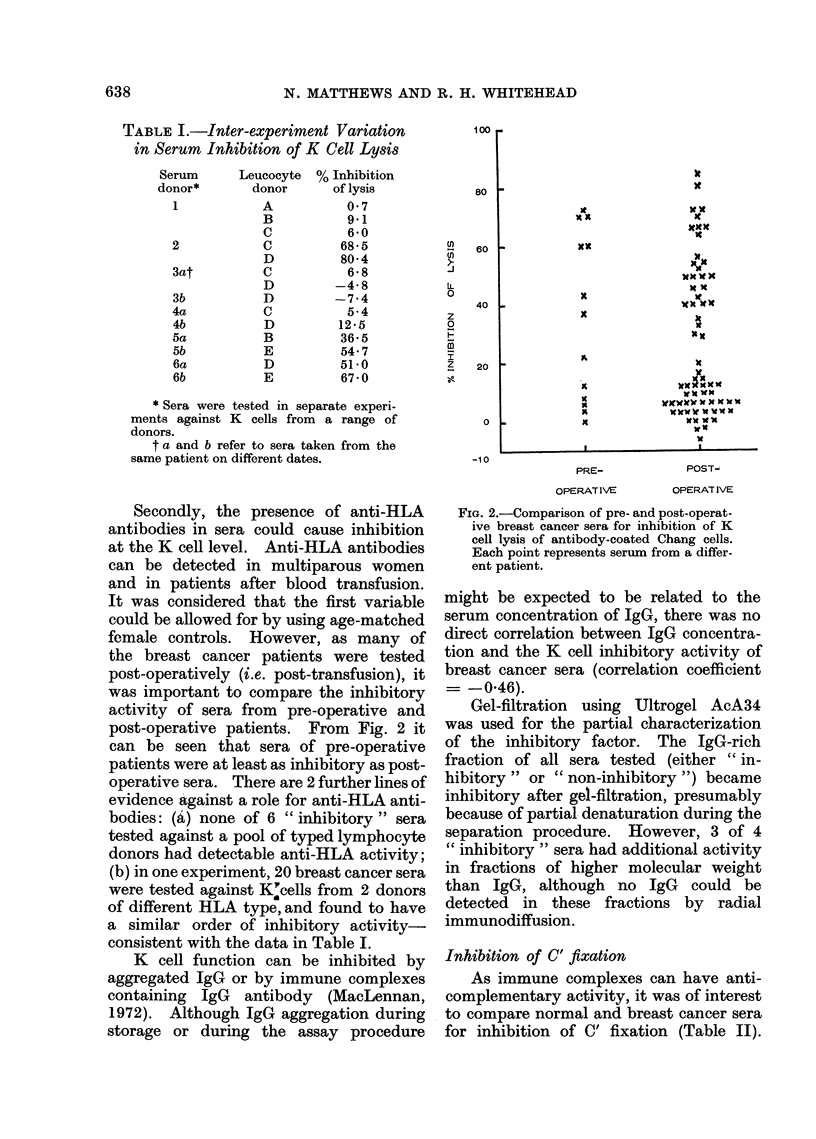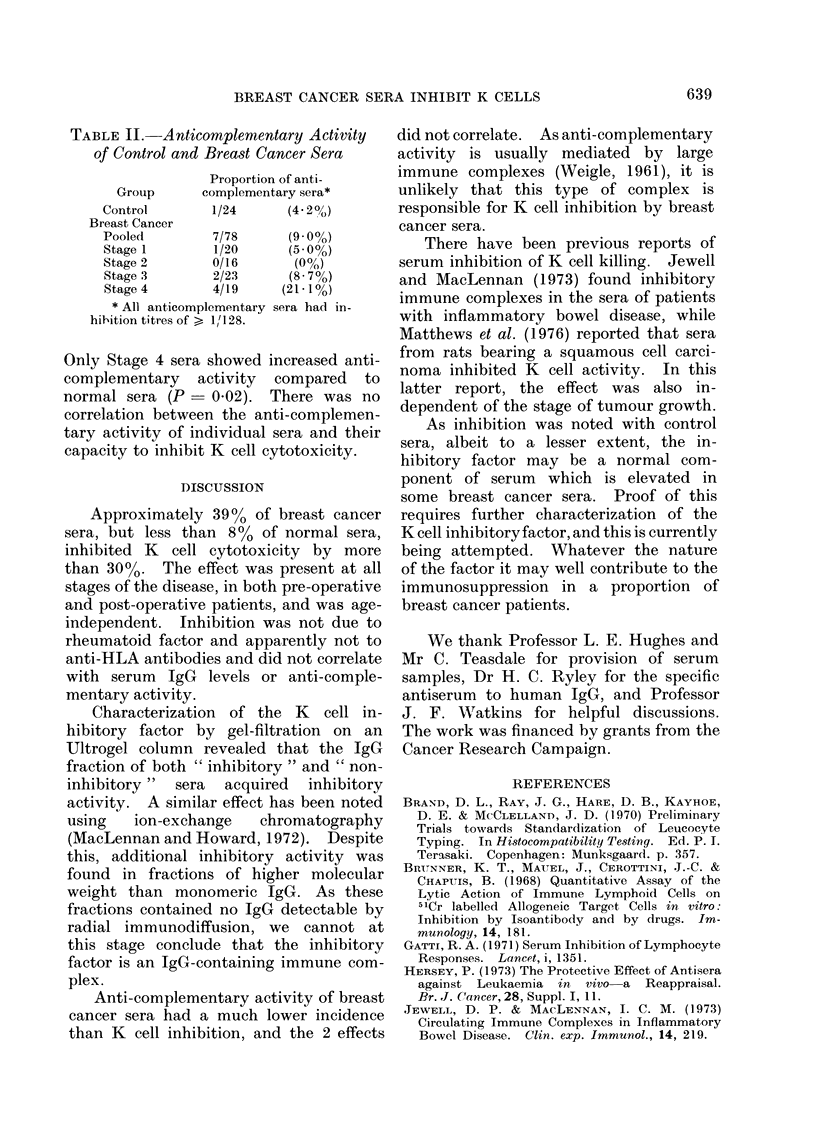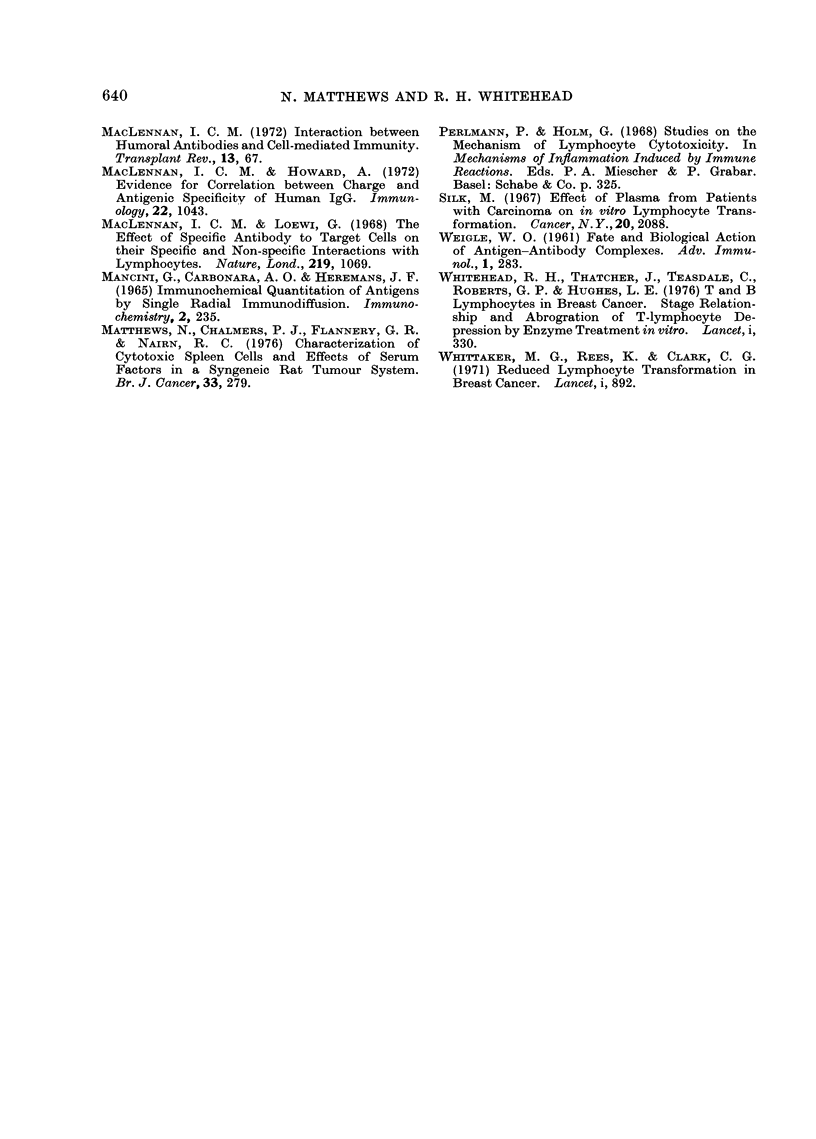# Inhibition of K cell function by human breast cancer sera.

**DOI:** 10.1038/bjc.1976.224

**Published:** 1976-12

**Authors:** N. Matthews, R. H. Whitehead

## Abstract

Sera from breast cancer patients and from female controls were tested for inhibition of lysis of antibody-coated target cells by human leukocytes (K cells). Sera from 39% of breast cancer patients, but from only 8% of controls, inhibited lysis by more than 30%. This inhibition was unrelated to the stage of the disease, the patient's age or whether the patient was pre- or post-operative. Inhibition was apparently not due to anti-HLA antibodies and did not correlate with the IgG level or anti-complementary activity of the serum. On fractionation by gel-filtration, inhibitory activity was found in fractions of higher molecular weight than IgG. As no IgG could be detected in these fractions, inhibition is probably not due to immune complexes containing IgG antibody. The inhibitory factor may well contribute to the immunosuppressed status of a proportion of breast cancer patients.


*Br. J. Cancer* (1976) **34**, 635

### INHIBITION OF K CELL FUNCTION BY HUMAN BREAST CANCER SERA

N. MATTHEWS* AND R. H. WHITEHEAD†

*From the Departments of *Medical Microbiology and †Surgery, Welsh National School of Medicine, Cardiff*

Received 11 June 1976 Accepted 28 July 1976

**Summary.—Sera from breast cancer patients and from female controls were tested for inhibition of lysis of antibody-coated target cells by human leucocytes (K cells). Sera from 39% of breast cancer patients, but from only 8% of controls, inhibited lysis by more than 30%. This inhibition was unrelated to the stage of the disease, the patient's age or whether the patient was pre- or post-operative. Inhibition was apparently not due to anti-HLA antibodies and did not correlate with the IgG level or anti-complementary activity of the serum. On fractionation by gel-filtration, inhibitory activity was found in fractions of higher molecular weight than IgG. As no IgG could be detected in these fractions, inhibition is probably not due to immune complexes containing IgG antibody. The inhibitory factor may well contribute to the immunosuppressed status of a proportion of breast cancer patients.**

MANY cancer patients have impaired immunological competence, especially later in the course of their disease. The basis of this impairment remains largely unknown, although sera from patients with widely different cancers can depress T lymphocyte function *in vitro* as measured by PHA stimulation (Silk, 1967; Whittaker, Rees and Clark, 1971; Gatti, 1971). Sera from breast cancer patients can also reduce E rosette formation, another T lymphocyte property (Whitehead *et al.*, 1976). However, the effect of cancer serum on the function of other types of lymphoid cell appears to have been relatively unexplored. In this study, sera from breast cancer patients have been tested for inhibition of K cell function. K cells are non-phagocytic lymphoid cells responsible for killing antibody-coated target cells (Perlmann and Holm, 1968; MacLennan and Loewi, 1968). This mechanism is potentially of importance in tumour rejection (Hersey, 1973).

### MATERIALS AND METHODS

*Patients.*—Patients ranged in age between 37 and 83 years (mean $\pm$ s.d., 59·8 $\pm$ 12·2) and were attending an outpatients breast cancer clinic. None of these women had received chemo- or radiotherapy in the previous 12 months. All patients were staged using clinicopathological data according to the TNM classification; Stage 1, $T_{1-2}N_0M_0$; Stage 2, $T_{1-2}N_1M_0$; Stage 3, $T_{3-4}N_{0-2}M_0$; Stage 4, $T_{1-4}N_{0-3}M_1$ where T, N and M represent respectively tumour size, the number of lymph nodes involved, and whether or not there is metastasis. The mean ages of the patients in the 4 stages were: Stage 1, 60·2 $\pm$ 13·9; Stage 2, 60·1 $\pm$ 12·6; Stage 3, 59·7 $\pm$ 11·5; Stage 4, 57·9 $\pm$ 12·0.

*Controls.*—Controls were healthy women, mostly laboratory or office staff aged between 41 and 72 years (mean 53·3 $\pm$ 7·9).

*Sera.*—Blood was obtained by venepuncture, allowed to clot at room temperature for 30 min and kept at 4°C for 2 h before collection of the serum by centrifugation. Sera were either stored at 4°C and used within 1 week of collection, or stored

Correspondence to: Dr N. Matthews, Department of Medical Microbiology, Welsh National School of Medicine, Heath Park, Cardiff CF4 4XN, Wales, U.K.

at −20°C until required. To avoid aggregation of IgG, sera were not heat-activated, but left overnight at room temperature before assay to remove the bulk of the complement activity.

*Inhibition of K cell killing.*—Sera were tested for their capacity to inhibit lysis of antibody-coated Chang target cells by K cells (MacLennan and Loewi, 1968). Normal human leucocytes from laboratory personnel were used as the source of K cells.

Peripheral blood leucocytes were obtained from heparinized blood by Hypaque/Ficoll centrifugation and washed × 3. Although the leucocyte suspension contained up to 15% monocytes and polymorphs, these cells did not lyse antibody-coated Chang cells—as shown by separate experiments using plastic adherence to remove the phagocytic cells.

Chang liver cells ($10^6$) were labelled with $^{51}$Cr (Brunner *et al.*, 1968), incubated for 30 min at 37°C with 1 ml of a 1/500 dilution of rabbit anti-human fibroblast serum, and washed twice. This is a sub-complement-fixing amount of antiserum. The antiserum was raised by giving 3 weekly injections of $10^6$ cells and bleeding 1 week after the last injection.

Sera (25 $\mu$l) were dispensed into the wells of Microtest II plates (Falcon Plastics) and 75 $\mu$l of a human leucocyte suspension ($1{\cdot}5 \times 10^5$ cells) was added, followed by 100 $\mu$l of a suspension of $^{51}$Cr-labelled, antibody-coated Chang cells ($10^4$). After 17 h incubation at 37°C in 5% $CO_2$:95% air, 100 $\mu$l of supernatant was removed for $\gamma$ counting. Each test was set up in quadruplicate, using Eagle's minimum essential medium containing 10% foetal calf serum as diluent. Inhibition of cytotoxicity was calculated from the formula 100 (a–b)/(a–c) where a, b and c are the mean ct/min $^{51}$Cr released from antibody-coated Chang cells by respectively, leucocytes + medium, leucocytes + test serum, and medium alone. The %$^{51}$Cr released by medium alone or by leucocytes + medium ranged from 17% to 35% and from 80% to 95% respectively (10 experiments). The standard deviation of quadruplicate cultures was always less than 7% of the mean, and usually less than 5%. For all of the lymphocyte donors used, " spontaneous " killing of *non-antibody-coated* Chang cells was less than 10%, and hence this effect was not taken into account when calculating inhibition of lysis of *antibody-coated* Chang cells. In each test, sera from controls and from cancer patients were included, and the data from all tests were plotted in Fig. 1.

*Rheumatoid factor estimation.*—The Rose–Waaler method was employed. Sera were tested for agglutination of rabbit IgG-coated sheep erythrocytes.

Erythrocytes (1%, v/v) were incubated for 30 min at room temperature with an equal volume of rabbit anti-sheep erythrocyte serum (Wellcome Reagents), diluted 10 times beyond its haemagglutination titre. Equal volumes of the coated cells and test serum dilutions were incubated overnight at 4°C in round-bottomed microplates (Cooke Engineering). Results were expressed as the minimum serum dilution which agglutinated at least half of the cells. Before testing, all sera were absorbed with sheep erythrocytes.

*HLA typing.*—Sera were tested by the NIH lymphocytotoxicity technique (Brand *et al.*, 1970) against selected lymphocytes, covering the following HLA antigens: HLA-A 1, 2, 3, 9, 11, 28, 29, w30, w31, w32. HLA-B 5, 7, 8, 12, 14, 18, 27, w15, w16, w17, w21, w22, w35, w40. Typing was kindly performed by Mr C. Darke at the Welsh Regional Transfusion Centre, St Fagans.

*Estimation of IgG levels.*—The radial immunodiffusion method was used (Mancini, Carbonara and Heremans, 1965).

*Serum fractionation by gel-filtration.*—Sera (1 ml) were fractionated at room temperature on a ($1{\cdot}5 \times 90$) cm column of Ultrogel AcA34, equilibrated with sterile Dulbecco's phosphate-buffered saline, pH 7·5. A flow rate of 3 ml/h was used and 2-ml fractions were collected at 4°C. Appropriate fractions were pooled and sterilized using a 0·2-$\mu$m filter.

*Inhibition of complement (C′) fixation.*—Sera were tested for inhibition of C′-dependent lysis of antibody-coated sheep erythrocytes.

Thrice-washed erythrocytes (1 ml packed cells) were incubated for 45 min at 37°C with 50 ml of a 1/500 dilution of rabbit haemolysin (Wellcome Reagents) in C′ fixation buffer (Oxoid), washed once and resuspended at 2% (v/v) in C′ buffer containing 1% foetal calf serum. Fresh guinea-pig serum was used as C′ and titrated against an equal volume of sensitized erythrocytes in flat-bottomed Microtest II plates (Falcon Plastics). A C′ dilution of 8 minimum haemolytic units was used for inhibition tests. Equal volumes (50 μl) of test serum dilutions and C′ were incubated at 37°C for 30 min. Inhibition was expressed as the lowest serum dilution which caused inhibition of haemolysis. Sera which inhibited at dilutions > 1 in 8 were considered anti-complementary.

*Statistical tests.*—The Fisher exact probability test was used.

### RESULTS

### *Inhibition of lysis by K cells*

Fig. 1 compares the capacity of sera from normal individuals and from cancer patients to inhibit lysis of antibody-coated Chang cells by K cells. Most of the normal sera inhibited by less than 30%, with only 2 out of 26 sera (8%) above this limit. Breast cancer sera exhibited a wider range of inhibition, with clusters of values below and above 30%. Of the total of 84 breast cancer sera tested, 33 (*i.e.* 39%) inhibited by more than 30%. When the patient sera were grouped into stages, the proportions which inhibited by more than 30% were: Stage 1, 9 of 23 (39%); Stage 2, 6 of 17 (35%); Stage 3, 13 of 28 (46%); Stage 4, 5 of 16 (31%). By the Fisher exact probability test, there is significantly higher inhibition than normal sera ($P < 0{\cdot}05$) by Stage 1, 2 and 3 sera and by pooled breast cancer sera: for Stage 4, $P = 0{\cdot}053$. Inhibition did not correlate with the age of the patient (correlation coefficient = 0·12).

The reproducibility of the assay is shown in Table 1. Individual sera tested on different occasions against different K cell donors had comparable activity. In addition, sera taken from the same individual at different times and tested on different occasions gave similar results.

This inhibition of K cell killing may be due to a number of mechanisms. Firstly, IgM rheumatoid factor in the test serum could inhibit, by masking the Fc part of the anti-Chang cell IgG antibody (IgM does not bind to K cells). To investigate this, inhibitory sera (*i.e.* >30% inhibition) were tested for rheumatoid factor (RF) by the Rose–Waaler test. Of 21 inhibitory cancer sera tested, only 2 had detectable RF activity.

FIG. 1.—Comparison of normal and breast cancer sera for inhibition of K cell lysis of antibody-coated Chang cells. Each point represents serum from a different individual.

TABLE I.—*Inter-experiment Variation in Serum Inhibition of K Cell Lysis*

| Serum donor* | Leucocyte donor | % Inhibition of lysis |
|---|---|---|
| 1 | A | 0·7 |
| | B | 9·1 |
| | C | 6·0 |
| 2 | C | 68·5 |
| | D | 80·4 |
| 3*a*† | C | 6·8 |
| | D | −4·8 |
| 3*b* | D | −7·4 |
| 4*a* | C | 5·4 |
| 4*b* | D | 12·5 |
| 5*a* | B | 36·5 |
| 5*b* | E | 54·7 |
| 6*a* | D | 51·0 |
| 6*b* | E | 67·0 |

* Sera were tested in separate experiments against K cells from a range of donors.

† *a* and *b* refer to sera taken from the same patient on different dates.

FIG. 2.—Comparison of pre- and post-operative breast cancer sera for inhibition of K cell lysis of antibody-coated Chang cells. Each point represents serum from a different patient.

Secondly, the presence of anti-HLA antibodies in sera could cause inhibition at the K cell level. Anti-HLA antibodies can be detected in multiparous women and in patients after blood transfusion. It was considered that the first variable could be allowed for by using age-matched female controls. However, as many of the breast cancer patients were tested post-operatively (*i.e.* post-transfusion), it was important to compare the inhibitory activity of sera from pre-operative and post-operative patients. From Fig. 2 it can be seen that sera of pre-operative patients were at least as inhibitory as post-operative sera. There are 2 further lines of evidence against a role for anti-HLA antibodies: (a) none of 6 " inhibitory " sera tested against a pool of typed lymphocyte donors had detectable anti-HLA activity; (b) in one experiment, 20 breast cancer sera were tested against K cells from 2 donors of different HLA type, and found to have a similar order of inhibitory activity—consistent with the data in Table I.

K cell function can be inhibited by aggregated IgG or by immune complexes containing IgG antibody (MacLennan, 1972). Although IgG aggregation during storage or during the assay procedure might be expected to be related to the serum concentration of IgG, there was no direct correlation between IgG concentration and the K cell inhibitory activity of breast cancer sera (correlation coefficient = −0·46).

Gel-filtration using Ultrogel AcA34 was used for the partial characterization of the inhibitory factor. The IgG-rich fraction of all sera tested (either " inhibitory " or " non-inhibitory ") became inhibitory after gel-filtration, presumably because of partial denaturation during the separation procedure. However, 3 of 4 " inhibitory " sera had additional activity in fractions of higher molecular weight than IgG, although no IgG could be detected in these fractions by radial immunodiffusion.

*Inhibition of C′ fixation*

As immune complexes can have anti-complementary activity, it was of interest to compare normal and breast cancer sera for inhibition of C′ fixation (Table II).

TABLE II.—*Anticomplementary Activity of Control and Breast Cancer Sera*

| Group | Proportion of anti-complementary sera* | |
|---|---|---|
| Control | 1/24 | (4·2%) |
| Breast Cancer | | |
| Pooled | 7/78 | (9·0%) |
| Stage 1 | 1/20 | (5·0%) |
| Stage 2 | 0/16 | (0%) |
| Stage 3 | 2/23 | (8·7%) |
| Stage 4 | 4/19 | (21·1%) |

* All anticomplementary sera had inhibition titres of ⩾ 1/128.

Only Stage 4 sera showed increased anti-complementary activity compared to normal sera ($P = 0{\cdot}02$). There was no correlation between the anti-complementary activity of individual sera and their capacity to inhibit K cell cytotoxicity.

### DISCUSSION

Approximately 39% of breast cancer sera, but less than 8% of normal sera, inhibited K cell cytotoxicity by more than 30%. The effect was present at all stages of the disease, in both pre-operative and post-operative patients, and was age-independent. Inhibition was not due to rheumatoid factor and apparently not to anti-HLA antibodies and did not correlate with serum IgG levels or anti-complementary activity.

Characterization of the K cell inhibitory factor by gel-filtration on an Ultrogel column revealed that the IgG fraction of both " inhibitory " and " non-inhibitory " sera acquired inhibitory activity. A similar effect has been noted using ion-exchange chromatography (MacLennan and Howard, 1972). Despite this, additional inhibitory activity was found in fractions of higher molecular weight than monomeric IgG. As these fractions contained no IgG detectable by radial immunodiffusion, we cannot at this stage conclude that the inhibitory factor is an IgG-containing immune complex.

Anti-complementary activity of breast cancer sera had a much lower incidence than K cell inhibition, and the 2 effects did not correlate. As anti-complementary activity is usually mediated by large immune complexes (Weigle, 1961), it is unlikely that this type of complex is responsible for K cell inhibition by breast cancer sera.

There have been previous reports of serum inhibition of K cell killing. Jewell and MacLennan (1973) found inhibitory immune complexes in the sera of patients with inflammatory bowel disease, while Matthews *et al.* (1976) reported that sera from rats bearing a squamous cell carcinoma inhibited K cell activity. In this latter report, the effect was also independent of the stage of tumour growth.

As inhibition was noted with control sera, albeit to a lesser extent, the inhibitory factor may be a normal component of serum which is elevated in some breast cancer sera. Proof of this requires further characterization of the K cell inhibitory factor, and this is currently being attempted. Whatever the nature of the factor it may well contribute to the immunosuppression in a proportion of breast cancer patients.

We thank Professor L. E. Hughes and Mr C. Teasdale for provision of serum samples, Dr H. C. Ryley for the specific antiserum to human IgG, and Professor J. F. Watkins for helpful discussions. The work was financed by grants from the Cancer Research Campaign.

### REFERENCES

BRAND, D. L., RAY, J. G., HARE, D. B., KAYHOE, D. E. & McCLELLAND, J. D. (1970) Preliminary Trials towards Standardization of Leucocyte Typing. In *Histocompatibility Testing*. Ed. P. I. Terasaki. Copenhagen: Munksgaard. p. 357.

BRUNNER, K. T., MAUEL, J., CEROTTINI, J.-C. & CHAPUIS, B. (1968) Quantitative Assay of the Lytic Action of Immune Lymphoid Cells on $^{51}$Cr labelled Allogeneic Target Cells *in vitro*: Inhibition by Isoantibody and by drugs. *Immunology*, **14**, 181.

GATTI, R. A. (1971) Serum Inhibition of Lymphocyte Responses. *Lancet*, i, 1351.

HERSEY, P. (1973) The Protective Effect of Antisera against Leukaemia *in vivo*—a Reappraisal. *Br. J. Cancer*, **28**, Suppl. I, 11.

JEWELL, D. P. & MACLENNAN, I. C. M. (1973) Circulating Immune Complexes in Inflammatory Bowel Disease. *Clin. exp. Immunol.*, **14**, 219.

MacLennan, I. C. M. (1972) Interaction between Humoral Antibodies and Cell-mediated Immunity. *Transplant Rev.*, **13**, 67.

MacLennan, I. C. M. & Howard, A. (1972) Evidence for Correlation between Charge and Antigenic Specificity of Human IgG. *Immunology*, **22**, 1043.

MacLennan, I. C. M. & Loewi, G. (1968) The Effect of Specific Antibody to Target Cells on their Specific and Non-specific Interactions with Lymphocytes. *Nature, Lond.*, **219**, 1069.

Mancini, G., Carbonara, A. O. & Heremans, J. F. (1965) Immunochemical Quantitation of Antigens by Single Radial Immunodiffusion. *Immunochemistry*, **2**, 235.

Matthews, N., Chalmers, P. J., Flannery, G. R. & Nairn, R. C. (1976) Characterization of Cytotoxic Spleen Cells and Effects of Serum Factors in a Syngeneic Rat Tumour System. *Br. J. Cancer*, **33**, 279.

Perlmann, P. & Holm, G. (1968) Studies on the Mechanism of Lymphocyte Cytotoxicity. In *Mechanisms of Inflammation Induced by Immune Reactions*. Eds. P. A. Miescher & P. Grabar. Basel: Schabe & Co. p. 325.

Silk, M. (1967) Effect of Plasma from Patients with Carcinoma on *in vitro* Lymphocyte Transformation. *Cancer, N.Y.*, **20**, 2088.

Weigle, W. O. (1961) Fate and Biological Action of Antigen–Antibody Complexes. *Adv. Immunol.*, **1**, 283.

Whitehead, R. H., Thatcher, J., Teasdale, C., Roberts, G. P. & Hughes, L. E. (1976) T and B Lymphocytes in Breast Cancer. Stage Relationship and Abrogration of T-lymphocyte Depression by Enzyme Treatment *in vitro*. *Lancet*, i, 330.

Whittaker, M. G., Rees, K. & Clark, C. G. (1971) Reduced Lymphocyte Transformation in Breast Cancer. *Lancet*, i, 892.